# Characteristics of parks associated with depression in women only: a cross-sectional study of 329,363 adults

**DOI:** 10.1186/s12916-026-04641-1

**Published:** 2026-01-21

**Authors:** Jonathan R. Olsen, Natalie Nicholls, Fiona M. Caryl, Thomas Astell-Burt, Jill P. Pell, Donald M. Lyall, Frederick K. Ho, Xiaoqi Feng, Richard Mitchell

**Affiliations:** 1 Institute for Social Science Research, The University of Queensland, Brisbane, QLD 4072 Australia; 2 School of Health & Wellbeing, University of Glasgow, Scotland, UK; 3 School of Architecture, Design and Planning, University of Sydney, Sydney, Australia; 4 Population Wellbeing and Environment Research Lab (PowerLab), Sydney, Australia; 5 Westmead Applied Research Centre, Westmead Hospital, Sydney, Australia; 6 Charles Perkins Centre, University of Sydney, Sydney, Australia; 7 Sydney Environment Institute, University of Sydney, Sydney, Australia; 8 School of Population Health, University of New South Wales (UNSW), Sydney, Australia

**Keywords:** Park, Urban green space, Green infrastructure, Depression, Mental health, Sex, Gender

## Abstract

**Background:**

To examine associations between public park characteristics within different walking distances from residential locations and depression, to distinguish between features within parks (e.g. amenities, attractions, facilities, tree cover) and park metrics in the home area (e.g. number of parks, size, and total area), and to employ rigorous geospatial analysis linking the best available objectively measured park and urban green space (UGS) exposures to validated depression outcomes across multiple scales.

**Methods:**

This population-based cross-sectional study utilised baseline data from 329,363 UK Biobank participants resident in urban areas. Prevalent diagnosed depression was defined as an ICD-10 code of F32 (depressive episode) or F33 (recurrent depressive disorder). Park characteristics and urban green space data were derived from Ordnance Survey Great Britain datasets and spatially linked to participants’ residential addresses. Three definitions of Home Catchment Area size were tested for every individual respondent: 400 m (m), 800 m, and 1600 m, as proxies for a 10-,20- and 40-min return walk respectively. Logistic regression models assessed associations with robust statistical approaches including assessment of interaction, correction for multiple testing, confounder adjustment, and sensitivity analyses.

**Results:**

Specific park characteristics within 20-min and 40-min catchments were associated with reduced depression likelihood among women only. Within 40-min catchments, protective associations were observed for recreational amenities (cafés: odds ratio (OR) 0.89, 95% confidence interval (CI) 0.85–0.93; toilets: *OR* 0.85, 95% *CI* 0.79–0.91), attractions (*OR* 0.83, 95% *CI* 0.80–0.87), sports facilities (*OR* 0.84, 95% *CI* 0.79–0.90), and tree canopy coverage (e.g. > 20%, *OR* 0.88, 95% *CI* 0.85–0.91). In a 20-min catchment, each 1% increase in urban greenspace classified as parks was associated with 11% reduced depression odds among women (*OR* 0.89, 95% *CI* 0.82–0.95). No significant protective associations were observed among men, with some paradoxical adverse associations identified.

**Conclusions:**

This study provides robust evidence for protective associations between park characteristics and depression among women, but not men. Findings support proximity-based planning concepts but challenge the current policy and practice focus on 20-min neighbourhood and identify park features which optimise preventive potential. Results have direct implications for evidence-based urban planning policy internationally, providing a framework for developing mental health-supporting green infrastructure that recognises sex-based differences.

**Supplementary Information:**

The online version contains supplementary material available at 10.1186/s12916-026-04641-1.

## Background

Evidence highlights the beneficial impact of greenspace on mental health, across diverse populations and contexts [1–4]. This evidence is extensive; one umbrella review consolidated findings from 40 systematic reviews [5]. Whilst multiple mechanisms through which greenspace might influence health have been identified and explored [6–9], three pathway domains have been highlighted as particularly beneficial for mental health (building capacities, restoring capacities, reducing harms), with one potentially detrimental domain operating via physical isolation and reducing walkability [8].

Research has examined associations between greenspace and multiple mental health outcomes, including depression, anxiety, mental illness, and mental wellbeing (both eudaimonic outcomes, such as personal flourishing, and hedonic outcomes, such as life satisfaction). Systematic reviews of these outcomes have yielded mixed findings, though evidence generally supports positive associations with mental wellbeing [10] and reduced risk of depression and anxiety [11]. In a review examining pathways linking greenspace to various mental health outcomes, Zhang and Zhang [6] identified considerable variation in the strength and nature of associations across different outcomes. Regarding depression—the focus of the present study—evidence suggests a direct relationship between greenspace exposure and depressive symptoms. Liu and Chen [11] noted that relatively few studies have investigated the impact of greenspace on depression and called for future research examining how different greenspace metrics relate to depression outcomes. The existing evidence indicates that relationships between greenspace and mental health vary according to the specific outcome examined, operating through different pathways. For depression specifically, findings suggest a direct association whereby greater greenspace exposure relates to fewer depressive symptoms [6], though further research is needed to clarify which specific greenspace characteristics are most relevant.

Researchers have quantified exposure to different forms of greenspace through various metrics [12–14]. Parks, defined as managed public areas designated for recreational use [13], are one key form of greenspace that may support mental health [15]. Research has predominantly explored the relationship between mental health and overarching neighbourhood or catchment park metrics capturing availability (e.g. the presence, number, and/or size of parks within specific residential proximity boundaries) [16]. However, given the variety of mechanisms by which parks might affect mental health, it is probable that different kinds of parks, with different specific characteristics, influence mental health to varying extents and via distinct causal mechanisms [17].

A growing body of evidence has examined associations between specific park amenities and mental health outcomes. Where parks were categorised into three functional types—recreation, sport, and nature spaces—all three conferred mental health benefits, suggesting multiple pathways through which park environments may support psychological wellbeing [18]. Additional research found that residential proximity to parks with sports amenities was associated with significantly greater improvements in mental health compared to parks lacking such facilities [19]. Using composite scores, parks with greater scores for “activity” and overall amenities were associated with enhanced mental health [20]. However, recent systematic reviews have highlighted insufficient evidence regarding which specific attributes, and composition of parks, might maximise health benefits, including amenity features, tree cover, and accessibility. Such evidence is needed to guide evidence-based planning, design, and management decisions [10]. One review found preliminary evidence that park typologies, vegetation diversity, and spatial characteristics such as size and connectivity correlate differently with health outcomes, with effects also moderated by demographic factors including sex and age [21]. However, the authors emphasised the need for comprehensive, nationwide studies linked to robust mental health measurements. It has been also recommended that research focusing on the characteristics of parks and health should employ objective, replicable measurement of park characteristics to maximise comparability both within and between nations [17].

Therefore, whilst parks hold potential for positive influence on mental health, a substantial research gap exists regarding which specific characteristics matter most and for whom. This study aims to address that gap by examining the relationship between objective and replicable park measurements of features (amenities, attractions, facilities, tree cover) and catchment metrics (number, size, and total area covered by parks) and depression prevalence. We also assess whether associations vary by proximity to parks with specific characteristics within 10-, 20-, and 40-min return walking distances from residential locations.

The study was designed with two broad hypotheses in mind.

- *Hypothesis 1* (distance-dependent effects): The strength of associations between park features and depression will vary by distance from home, with associations diminishing as distance increases. Additionally, we hypothesise that larger parks may exhibit protective associations over greater distances than smaller parks, reflecting their capacity to draw users from a broader catchment area.
- *Hypothesis 2* (amenity-specific mechanisms): The presence of specific park amenities that support usability and social interaction (toilets, attractions, recreational facilities) will explain variance in depression outcomes beyond that accounted for by basic park proximity and size alone, indicating that park quality and functionality matter independently of accessibility.

## Methods

### Study design and participants

A cross-sectional study (*n* = 329,363) was conducted using data from the UK Biobank cohort study, which contains individual-level sociodemographic and health information. The original Biobank sample was 502,366 UK residents. Participants were recruited between 2006 and 2010 from England, Scotland, and Wales, aged 37–73 years. All participants attended 1 of 22 assessment centres, where they undertook face-to-face interviews, completed a self-administered touchscreen questionnaire, and underwent physical examination by trained staff. Participants’ health behaviours (e.g. smoking) were self-reported using validated survey measures. UK Biobank has ethics approval from the North-West Multicentre Research Ethics Committee (Reference 16/NW/0274), and this study was conducted under project 73613.

### Measurements

#### Outcome variable: depression

The mental health outcome was a history of depression on recruitment to UK Biobank, ascertained through either self-report or retrospective linkage to primary and secondary care records. The measure records depression prevalence at baseline. It was selected over subsequent incident depression since within UK Biobank, incident depression is based solely on hospitalisations, rendering it relatively underpowered and capturing only the extreme end of severity. Additionally, depression is among the most prevalent mental health conditions globally, with incidence rates continuing to rise [22]. Depression represents a major public health priority due to its well-established associations with premature mortality and comorbid physical illnesses [23]. When completing the baseline questionnaire, Biobank participants reported conditions which had ever been diagnosed by a physician, and these were then verified by a trained nurse during a face-to-face interview. In addition, retrospective record linkage to primary and secondary care records was used to identify ICD-10 codes or F32 (depressive episode) or F33 (recurrent depressive disorder).

#### Exposure variables: parks and urban green space (UGS)

**Fig. 1:**
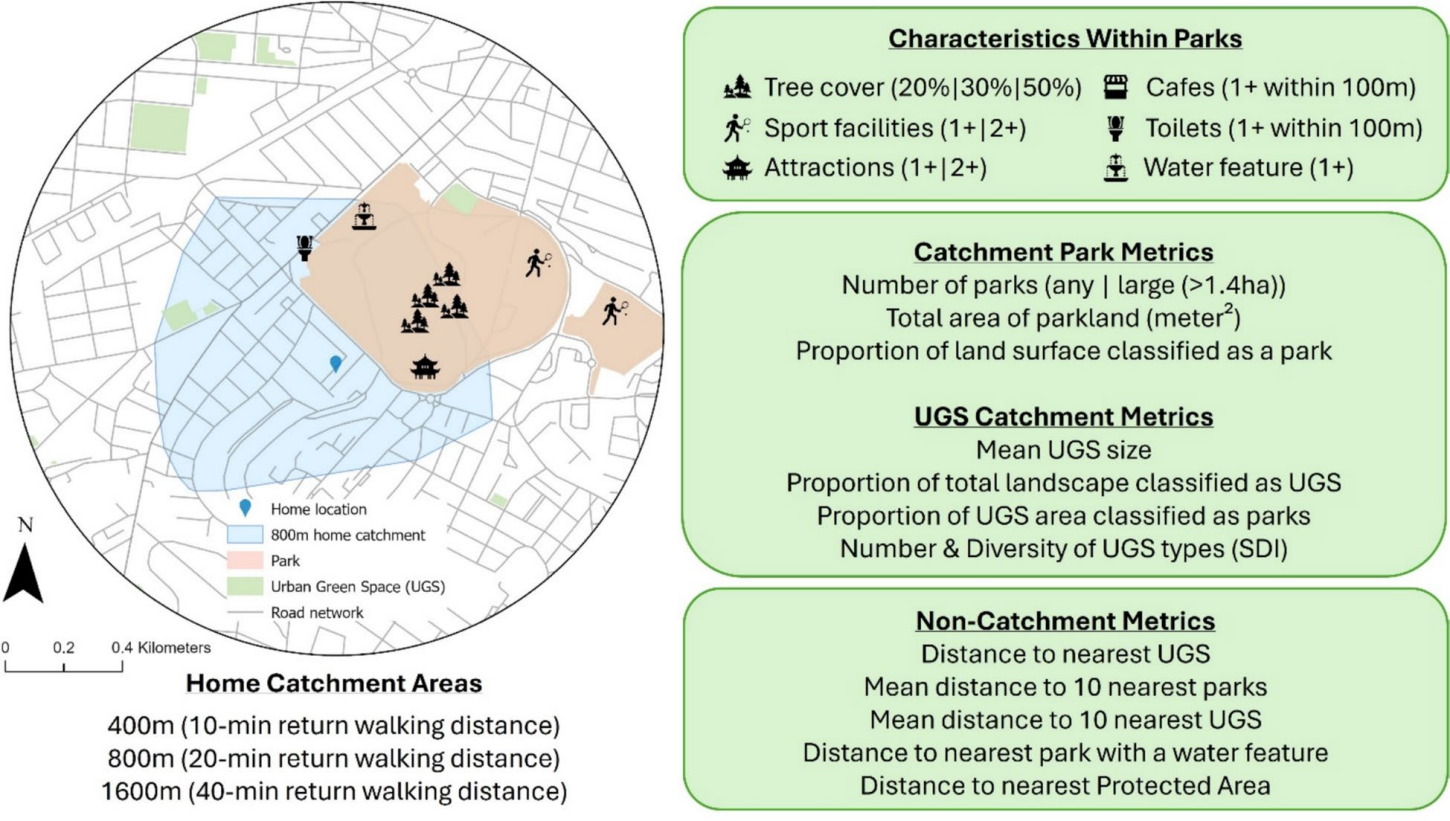
Park and urban green space (UGS) variables

Assessing the characteristics of parks to which Biobank participants might be exposed was carried out in three key stages (Fig. 1).

In step 1, *Home Catchment Areas* were created based on an 800-m (m) road and path network distance from each UK Biobank participant’s residential location. The 800-m catchment area was chosen based on a 20-min return walking distance from home. This distance corresponds to a “20-min neighbourhood”, currently a unit of analysis for neighbourhood living with global resonance and policy currency [24, 25]. For sensitivity analysis and to explore hypothesis 1, catchment areas were also then calculated for 400-m and 1600-m distances.

In step 2, the features and content of all parks within the Home Catchment Area of each Biobank participant were assessed. We labelled these variables *Characteristics Within Parks*. These enabled the assessment of hypothesis 2. The features and content which comprised Characteristics Within Parks were based on a previously developed in situ audit tool [26] that was adapted for use with digital and remotely sensed data, enabling assessment of all parks in the UK without the need for a physical survey of each one. A detailed description of the creation of Characteristics Within Parks has been given previously [27, 28]. In brief, the measure utilised digitised spatial data from Ordnance Survey to identify and count the amenities (cafés and toilets inside the park or within 100 m of its boundary), attractions (e.g. botanical gardens, historical sites), sports facilities (e.g. tennis courts), water features (moving or standing water), and tree cover, within each park (Fig. 1). The presence of amenities, attractions, sports facilities, and water features was categorised per park based on whether they contained zero, one or more, or two or more. Tree cover was categorised as > 20%, > 30%, and > 50% of parks being covered by trees (thresholds selected on the basis of previous work [29]). Finally, the Characteristics Within Parks were aggregated within each Biobank participant’s 800-m Home Catchment Area (repeated for the 400-m and 1600-m sensitivity analyses). Details of the Within Park Characteristics, their definition and source are provided in Additional File 1: Table S1.

In step 3, we calculated *catchment park metrics*. These quantified aspects of the availability of parks in general within each Biobank participant’s Home Catchment Area. The metrics were the number of parks, the numbers of large parks (> median 1.4 ha), the total area of parkland, and proportion of land surface classified as a park, within the Home Catchment Area.

Parks are not the only form of greenspace available in an urban area. Other kinds of urban greenspace, and total greenspace, have also been associated with mental health [29, 30]. We therefore also created measures of all urban greenspaces within Home Catchment Areas to include as potential confounders. We used the Ordnance Survey definition that includes greenspaces which are likely to be accessible to the public, such as playing fields, sports facilities, play areas, and allotments [31]. Our urban green spaces (UGS) *catchment metrics* were mean UGS size (m^2^) within the Home Catchment Area, proportion of Home Catchment Area covered by UGS, proportion of UGS within catchment specifically classified as a park, and number and diversity (Shannon Diversity Index (SDI)) of UGS types within the Home Catchment Area (capturing, for example, the balance between formal recreational spaces and other green infrastructure).

Finally, acknowledging that a fixed distance might not appropriately capture the neighbourhood or activity space for a Biobank participant, we computed a set of exposure measures that were not tied to the Home Catchment Area. We labelled these *non-catchment metrics*, and they were as follows: distance to nearest UGS (m), mean distance to 10 nearest parks (m), mean distance to 10 nearest UGS, distance to nearest UGS with a water feature (m), and distance to nearest protected (i.e. officially recognised for nature or biodiversity value) area (m).

#### Confounders

Based on existing literature [17, 32, 33], a number of individual-level confounders were chosen to include as covariates: age, sex, ethnicity (White or Black, Asian, and Minority Ethnic (BAME)), household income, education, employment status, and area-level deprivation. Area-level deprivation was based on the Index of Multiple Deprivation (IMD) for each GB nation, which assess the extent to which an area is deprived across a number of domains [34]. We used the income domain only as the overall indicator also includes measures of proximity to amenities and health outcomes, which are indicators of interest for our study.

The following variables were also explored as covariates in a sensitivity analysis (Additional File 1: Table S2a and b): alcohol frequency, smoking, body mass index (BMI), physical activity, and self-reported history or diabetes and hypertension. These variables were included as they have been shown to be potentially associated with both mental health and residential location (and therefore access to parks and UGS [32, 33]). However, other studies have identified that they are also potentially mediators and/or moderators in the relationship between parks and depression. Given statistical advice that adjustment for these could add bias rather than reduce it, they were not included in the model presented in the main results.

### Statistical analyses

The sample of UK Biobank respondents we analysed was restricted to those who resided within an urban area (England/Wales: “urban — less sparse”; Scotland: “large urban area”, “other urban area”). This enabled use of the Ordnance Survey UGS variable which focuses on urban areas only. Given that the outcome was binary (depression yes/no), logistic regression was used for the models, adjusting for the confounders detailed above. We tested for, but did not observe, moderation of associations by area-level socio-economic position. However, we did observe statistically significant interactions between sex and the exposures of interest (Additional File 1: Table S3), and the analyses we present are stratified by sex. Given that the exposure variables capture characteristics present (or not) in parks, the analysis for catchment-based features was restricted to those who had a park within each of the catchments.

Primary care is the most common origin of a routinely recorded diagnosis of depression, and it is plausible that regional variation in recording of diagnoses might have introduced bias in prevalence. To assess this, sensitivity analyses were performed on a subset of participants which excluded those who had an ICD-10 code derived from primary care; no substantive differences in effect directions were found (Additional File 1: Table S4a and b), and we opted to retain diagnoses stemming from primary care.

Results are presented as odds ratios (OR) and 95% confidence intervals (CI). A global alpha of 5% was set, and a Bonferroni corrected *p*-value (0.05/20 = 0.0025) was used to determine “significance” for all analytical outputs. Data manipulation was performed in Stata 18 [35] and R 4.4.0 [36]; all analysis was performed in R.

## Results

### Sample

The total analysed sample consisted of 329,363 adults, 53% female (median age female: 57 years, male: 58 years) (Table 1). A higher proportion of females had a depression diagnosis (10%), compared to males (6%). There were no sex-based differences in having a park within 400-m, 800-m, or 1600-m home catchment areas.

**Table 1:** Sample characteristics

		**Female**	**Male**
Base sample		174,531	154,832
Age (median (IQR))		57 (50 to 57)	58 (51 to 59)
		%	
Depression	ICD code of F32/33	10.1	6.0
Park within home catchment (1 or more)	400 m	19.8	19.5
	800 m	49.0	47.9
	1600 m	78.6	77.9
Area-level income deprivation (quintiles)	Q1—least deprived	25.3	26.3
	Q2	18.5	18.7
	Q3	21.3	20.9
	Q4	19.1	18.4
	Q5—most deprived	15.8	15.7
Ethnicity	White	95.3	95.5
	Black, Asian, and Minority Ethnic (BAME)	4.7	4.5
Household income (£)	Less than 18,000	18.7	13.8
	18,001 to 30,999	26.1	23.5
	31,000 to 51,999	28.0	29.6
	52,000 to 99,999	21.6	26.0
	More than 100,000	5.6	7.2
Education	Higher education	40.5	42.9
	Other	59.5	57.1
Employment status	Employed	64.0	67.1
	Retired	28.1	27.9
	Other	5.2	0.9
	Unable to work	1.9	2.2
	Unemployed	0.9	1.9

### Base model and sex-based interaction effects

The base models (covariates only) are provided in Additional File 1: Table S5, and these models indicated that all variables tested were associated with depression, and multicollinearity testing was satisfactory (Generalised Variance Inflation Factor (GVIF), < 2, or adjusted GVIF < √2).

### Characteristics within parks, catchment park metrics, USG metrics, and depression

For the 800-m home catchment areas (a proxy for a 20-min return trip from home), numerous protective associations were found between characteristics within parks, catchment park metrics, UGS metrics, and depression but almost entirely among women only (Table 2). For characteristics within parks, toilets, attractions, and some levels of tree cover were associated with reduced risk among women. For catchment park metrics, both the number and the number of large parks showed a protective association among women. The proportion of catchment UGS classified as parks showed a relatively strong association for women with an 11% reduction in the odds of depression for a 1% increase in the proportion of UGS area classified as parks (*OR* 0.89, 95% *CI* 0.82–0.95). Not only results for men were largely not significant but also, surprisingly, associations were largely adverse—that is indicating greater odds of depression. For example, the presence of 1 + sports facilities within a park was positively associated with depression among men (*OR* 1.08, 95% *CI* 1.00–1.16).

**Table 2:** Association between park, UGS variables and depression (females)

Females		400-m catchment				800-m catchment				1600-m catchment			
		OR	LCL	UCL	*p*	OR	LCL	UCL	p	OR	LCL	UCL	*p*
Amenities	Café	0.98	0.91	1.06	0.63	0.97	0.92	1.03	0.38	0.89	0.85	0.93	< 0.001
	Toilet	1.00	0.92	1.08	0.92	0.94	0.89	1.00	0.05	0.85	0.79	0.91	< 0.001
Attractions	1 + attractions	0.93	0.86	1.00	0.05	0.90	0.85	0.95	< 0.001	0.83	0.80	0.87	< 0.001
	2 + attractions	0.91	0.85	0.99	0.02	0.94	0.88	0.99	0.02	0.85	0.79	0.91	< 0.001
Tree cover	20% of park area	0.99	0.91	1.08	0.88	0.93	0.88	0.99	0.02	0.88	0.85	0.91	< 0.001
	30% of park area	0.97	0.90	1.05	0.48	0.96	0.91	1.01	0.12	0.88	0.85	0.91	< 0.001
	50% of park area	0.96	0.89	1.03	0.25	0.95	0.90	1.00	0.07	0.87	0.83	0.91	< 0.001
Water feature		1.01	0.94	1.09	0.74	1.01	0.96	1.06	0.77	0.87	0.82	0.93	< 0.001
Sports facilities	1 + sports facility	0.98	0.91	1.05	0.59	0.96	0.91	1.02	0.20	0.84	0.79	0.90	< 0.001
	2 + sports facility	0.96	0.89	1.05	0.39	0.96	0.90	1.02	0.18	0.83	0.76	0.91	< 0.001
Catchment park metrics	Number of parks	0.98	0.90	1.06	0.58	0.98	0.96	1.00	0.05	0.98	0.97	0.98	< 0.001
	Number of large parks (> median 1.4 ha)	0.97	0.90	1.05	0.46	0.94	0.89	0.99	0.02	0.85	0.81	0.89	< 0.001
	Area (m) of catchment covered by parks	1.00	1.00	1.00	0.96	1.00	1.00	1.00	0.12	1.00	1.00	1.00	0.004
	Proportion of catchment covered by parks	1.15	0.73	1.78	0.55	0.75	0.51	1.10	0.14	0.36	0.25	0.52	< 0.001
UGS catchment metrics	Mean size of UGS within catchment	1.03	0.98	1.08	0.24	0.99	0.97	1.01	0.21	0.99	0.98	1.00	0.10
	Proportion of catchment UGS	1.30	0.91	1.83	0.14	1.04	0.78	1.38	0.78	0.63	0.49	0.82	< 0.001
	Proportion of UGS area classified as parks	0.92	0.82	1.02	0.12	0.89	0.82	0.96	< 0.001	0.76	0.71	0.82	< 0.001
	Number of types of UGS	1.01	0.98	1.05	0.59	1.01	0.98	1.03	0.63	1.00	0.97	1.02	0.89
	Shannon diversity of UGS types	1.07	0.97	1.18	0.17	1.05	0.98	1.12	0.16	1.10	1.03	1.17	0.01
**Non-catchment metrics**													
		**OR**			**LL CI**			**UL CI**			***P***		
Distance to nearest park		1.01			1.00			1.03			0.03		
Mean distance to 10 nearest parks		1.02			1.01			1.03			< 0.001		
Mean distance to 10 nearest UGS		0.97			0.92			1.03			0.33		
Distance to nearest park with a water feature		1.02			1.01			1.03			< 0.001		
Distance to nearest protected area		1.01			1.00			1.02			0.002		

### A 400-m home catchment and 1600-m catchment sensitivity analyses

Within the smaller 400-m catchment (10-min return trip from home), fewer no associations with depression were observed, but the general pattern of reduced odds for women and increased odds for men recurred (Tables 2 and 3). For the 1600-m home catchment (40-min return trip from home), all the characteristics within parks and catchment park metrics, and most of the UGS catchment metrics, were associated with a reduced likelihood of depression among women (Table 2), but the strength of protective association was broadly (and sometimes significantly) greater for the 1600-m catchment than the 800 m. The 1600-m distance threshold also saw the only significant protective association with depression among men, with park tree cover at 20% associated with reduced odds (*OR* 0.96, 95% *CI* 0.920–1.00).

**Table 3:** Association between UGS variables and depression (males)

Males		400-m catchment				800-m catchment				1600-m catchment			
		**OR**	**LCL**	**UCL**	***p***	**OR**	**LCL**	**UCL**	***p***	**OR**	**LCL**	**UCL**	***p***
Amenities	Café	1.03	0.92	1.14	0.63	1.03	0.95	1.11	0.50	1.00	0.95	1.06	0.92
	Toilet	1.06	0.95	1.18	0.27	1.09	1.00	1.18	0.04	1.05	0.96	1.15	0.26
Attractions	1 + attractions	0.99	0.89	1.09	0.82	1.02	0.95	1.09	0.64	0.98	0.93	1.03	0.40
	2 + attractions	1.02	0.92	1.13	0.67	1.06	0.98	1.14	0.13	1.08	0.98	1.18	0.13
Tree cover	20% of park area	1.02	0.91	1.13	0.78	1.01	0.93	1.09	0.87	0.96	0.92	1.00	0.05
	30% of park area	1.03	0.93	1.14	0.55	1.00	0.94	1.08	0.90	0.97	0.93	1.01	0.19
	50% of park area	1.01	0.91	1.11	0.91	1.00	0.93	1.07	0.97	0.96	0.91	1.01	0.09
Water feature		1.11	1.01	1.23	0.03	1.05	0.98	1.12	0.19	1.05	0.97	1.13	0.28
Sports facilities	1 + sports facility	0.94	0.86	1.04	0.24	1.08	1.00	1.16	0.04	1.05	0.96	1.14	0.27
	2 + sports facility	1.00	0.90	1.11	0.98	1.06	0.98	1.15	0.17	1.08	0.96	1.22	0.21
Park metrics	Number of parks	0.95	0.86	1.05	0.34	0.99	0.96	1.02	0.42	0.99	0.99	1.00	0.08
	Number of large parks (> median 1.4 ha)	1.14	1.02	1.26	0.02	1.02	0.94	1.09	0.68	0.98	0.92	1.04	0.48
	Area (m) of catchment covered by parks	1.00	1.00	1.00	0.06	1.00	1.00	1.00	0.11	1.00	1.00	1.00	0.75
	Proportion of catchment covered by parks	1.58	0.89	2.72	0.12	1.26	0.77	2.05	0.35	0.93	0.58	1.49	0.77
UGS metrics	Mean size of UGS within catchment	1.04	0.98	1.10	0.19	1.01	0.99	1.03	0.28	0.99	0.98	1.01	0.25
	Proportion of catchment UGS	1.30	0.82	2.04	0.26	1.22	0.84	1.75	0.29	0.94	0.67	1.33	0.73
	Proportion of UGS area classified as parks	1.17	1.01	1.36	0.04	1.00	0.90	1.10	0.94	0.98	0.89	1.08	0.75
	Number of types of UGS	0.99	0.94	1.03	0.59	1.01	0.98	1.04	0.64	1.00	0.97	1.04	0.89
	Shannon diversity of UGS types	0.97	0.85	1.10	0.61	0.98	0.89	1.07	0.60	1.03	0.94	1.12	0.56
**Non-catchment metrics**													
		**OR**			**LL CI**			**UL CI**			**P**		
Distance to nearest park		0.99			0.97			1.01			0.24		
Mean distance to 10 nearest parks		0.99			0.98			1.01			0.33		
Mean distance to 10 nearest UGS		0.94			0.87			1.01			0.11		
Distance to nearest park with a water feature		1.00			0.98			1.01			0.48		
Distance to nearest protected area		1.00			0.99			1.02			0.43		

### Non-catchment metrics

For women only, a greater mean distance to the nearest park and nearest 10 parks and distance to the nearest park with a water feature were associated with higher odds of having depression. No significant associations were observed among males.

Sensitivity analysis, adjusting for the further confounders detailed in the methods (alcohol frequency, smoking, BMI, physical activity, self-reported history or diabetes and hypertension), showed no substantive changes in results (Additional File 1: Table S2a and b).

## Discussion

### Summary of results

This study examined associations between park characteristics and prevalent depression by linking comprehensive spatial data on park features within a bespoke home catchment area to granular data on 329,363 UK Biobank participants. Depression was significantly more prevalent among female participants compared to male, in line with expectations and prior research [37], despite equivalent park availability within their home catchment areas.

We saw no evidence for moderation of associations by area-level socioeconomic deprivation. However, our study revealed a pattern of sex-based differences in associations between within park characteristics, catchment park metrics, UGS metrics, and risk of depression. In 800-m home catchment areas (equating to a 20-min neighbourhood), we identified numerous protective associations between park features and depression, but these were almost exclusively observed among women. Specific amenities including toilets, attractions, and moderate tree cover were associated with reduced depression risk for women, as was access to a greater number of parks, particularly larger parks. Most notably, the proportion of UGS classified as parks showed a substantial protective effect for women, with each 1% increase in park proportion associated with an 11% reduction in depression odds. These protective associations strengthened at the 1600-m catchment level, suggesting that women may benefit from park access across a broader geographical range than previously recognised.

In stark contrast, park characteristics showed largely nonsignificant or paradoxically adverse associations with depression among men. The presence of sports facilities within parks was associated with increased depression odds for men, and this pattern of counterintuitive findings persisted across most park metrics, albeit largely without statistical significance.

Our initial hypotheses were therefore largely confirmed; home catchment area did affect the strength of associations observed, and park amenities were important determinants of association.

### Comparison with existing literature

Previous research has documented sex-based differences in associations between greenspace and health [30, 33, 38]. A review examining sex and gender differences in urban greenspace and mental health explored various hypotheses for these disparities [39], including females’ greater likelihood of developing mental health conditions and potentially deriving greater greenspace benefits. The authors concluded that further research examining causal pathways for sex-based differences in greenspace protection is required. Our study, teasing out the influence of different park and urban green space qualities, begins to answer that call.

The sex-specific associations we observed between parks and depression may reflect fundamental differences in how men and women engage with and value these spaces. Evidence demonstrates that men and women utilise parks for distinct activities and prioritise different amenities. Men predominantly engage in physical activity at sports fields and gyms, while women more commonly use swimming pools and walking paths, with women exhibiting higher overall activity levels in parks [40]. Our study showed a positive association with sports facilities and depression for men, requiring further investigation of recent shifts in park utilisation by sex. Beyond activity patterns, women place greater value on park aesthetics and maintenance quality, factors previously linked to enhanced wellbeing [41]. A perceived stress study found negative (i.e. protective) associations with higher urban green space levels for both sexes, though females in low green space areas experienced greater stress [42]. When evaluating park features that influence visitation decisions, women rate safety and the presence of gardens as significantly more important than men do [43]. These preferences extend to social engagement patterns: women participate more frequently in social activities within parks and articulate specific, amenity-related reasons for park selection (e.g. “it has bathrooms” or “a good playground for my children”), whereas men often provide vague responses such as “I don't know” or “I was walking past” [44].

Several additional factors may explain why UGS characteristics were not associated with reduced depression among men in our study, warranting further investigation. First, depression manifests differently by sex: men commonly exhibit irritability, anger, and risk-taking behaviours rather than the low mood and social withdrawal more typical in women [45]. These masculine-typed symptoms may lead to different patterns of park disengagement or indicate that park characteristics operate through mechanisms less relevant to male depression phenotypes. Second, many of the UGS amenities we included facilitate social interaction and aesthetic quality—features that women value more highly than men [41]. Men may derive mental health benefits from different park attributes not captured in our framework, such as opportunities for vigorous physical activity, solitude, or unstructured use. If the amenities we measured do not align with the pathways through which men benefit from parks, null associations would be expected. Finally, unmeasured neighbourhood-level confounding may obscure associations among men.

Our findings contribute to the evidence base by demonstrating sex-based differences in park protective effects within 800-m and 1600-m catchments for females, though null findings [46, 47] and opposite-direction associations favouring males [48] have been reported elsewhere. Future research incorporating qualitative methods to examine gendered social norms, expectations, and barriers to park use would provide crucial insights into these disparate associations.

### Potential mechanisms and explanations

The mechanistic relationship between park attributes and mental health remains under-explored, though the pathway domains model [8] is helpful in generating hypotheses. Women’s stronger associations with park accessibility may reflect higher rates of park utilisation for activities that directly support mental health, such as social interaction or stress-reducing behaviours like walking or relaxation. Research consistently shows that women are more likely to use parks for social purposes and passive recreation, whereas men more commonly engage with parks for organised sports or physical exercise [40].

The presence of amenities and attractions within parks may facilitate these social aspects of green space use, strengthening social connections—a pathway that appears more important for women than men based on our findings. The protective effect of park amenities like toilets and attractions for women may reflect practical barriers that disproportionately affect female park usage, particularly for those with caregiving responsibilities or safety concerns. Greater choice and diversity in proximal parks may also encourage increased visitation frequency, with evidence suggesting that health improvements, including mental wellbeing, occur with at least two weekly visits [49]. This may explain why both the number of parks and the proportion of urban green space classified as parks showed protective associations for women in our study.

Tree canopy proportion within parks showed positive mental health associations, consistent with findings measuring tree canopy within home catchments [29, 50, 51]. Tree canopies demonstrate more protective effects across health outcomes compared to other greenspace types [52], hypothesised to result from promoting biodiverse landscapes associated with improved psychological wellbeing [29], as well as providing shaded areas that can act as meeting places and places of symbolic meaning to communities [53]. For example, a recent longitudinal study in Australia reported a 15% reduction in the odds of becoming lonely among women, but not men, for every 10% increase in tree canopy within 1.6 km [54], echoing the sex-based patterns observed in our study.

The paradoxical adverse associations observed among men, particularly the increased depression risk associated with sports facilities, may indicate that proximity to recreational infrastructure highlights unmet expectations or social pressures around physical activity and masculine identity. Alternatively, men experiencing depression may be less likely to utilise available park resources, creating a reverse causation whereby depressed men living near well-equipped parks feel more acutely aware of their reduced engagement with these facilities. The weak protective effects observed only at greater distances (1600 m) for men might hint that their relationship with green space operates through different spatial or social mechanisms, possibly involving different patterns of park utilisation—for example trips to access parks that are further away might mean more time spent there and hence longer or deeper engagement with nature.

### Implications for policy and practice

Our findings present both opportunities and challenges for urban planning and public health policy. The protective associations between specific park amenities and depression observed suggest that current park designs may inadvertently exclusively benefit women. To support the designing of urban parks to provide equitable health benefit between men and women, future studies should investigate whether men with depression are using parks and what modifications might support men’s mental health. Such evidence would support the development of targeted, equity-oriented policies that maximise mental health benefits across diverse populations while acknowledging that universal environmental interventions may have heterogeneous effects.

Existing evidence supports positive mental health benefits from greater numbers of parks within 1600-m buffers, particularly larger parks with recreational or sporting facilities [18]. Park amenities within larger catchments were more strongly linked to depression in our study for women, with protective associations strengthening in the 1600-m home catchment area. This suggests that national guidelines, such as Scotland’s National Performance Framework which measures green and blue space access within 5-min walks [55], should consider broadening their spatial distance measures to include 10-min (800 m) and 20-min (1600 m) walks, as our study provides evidence of depression prevention benefits at these distances.

In contrast to the positive associations observed for catchment-based metrics, we found that distance-based non-catchment metrics showed small associations with increased depression prevalence among women, including mean distance to the 10 nearest parks and distance to the nearest park with a water feature. This suggests that whilst local access matters, the broader spatial distribution of parks across the urban landscape also influences mental health outcomes. These distance metrics may capture broader neighbourhood characteristics, beyond parks, related to urban planning quality, social deprivation, or environmental justice that warrant further investigation. There are fundamental characteristics underpinning residential location patterns, such as residential self-selection, which also correlate with mental health [56] and showed mixed evidence for park use and subsequent physical activity, where perceived access to outdoor recreational facilities were most strongly associated with residential self-selection, compared to objective measures [57]. Studies have highlighted that controlling for residential self-selection—using three indicators of neighbourhood preference—reduces confounding by up to 44% [58]; indicating that future studies should employ strategies to control for this within their analysis by including measures of residential self-selection.

Our framework for evaluating UGS qualities has potential applicability across diverse global contexts, though several considerations are warranted. While most evidence examining relationships between greenspace and depression originates from high-income countries in the Global North, emerging research from the Global South demonstrates relevant patterns. For example, studies in Latin American cities have found associations between area-level greenspace and depression outcomes [59], though no relationship with specific park amenities. The transferability of this framework depends on several contextual factors, including the following: (1) local patterns of park usage, (2) cultural norms around public space, (3) climate and seasonal variation affecting park accessibility, and (4) socioeconomic factors that may moderate greenspace access and utilisation. Researchers applying this framework in different settings should adapt amenity classifications to reflect locally relevant features and account for context-specific confounders.

### Strengths and limitations

This study has several strengths, including the novel, detailed park and catchment characteristic variables, a large sample that includes a range of sociodemographic and health variables for inclusion. We controlled for key confounders, as well as total UGS within home catchments. Due to the number of significant tests, a higher significance threshold was used as a cutoff for assessing the association between park features and depression. The use of objective and replicable measures of park qualities and attributes in a national scale cohort analysis represents an important step forward. The international relevance of these findings is underscored using objective, standardised measures of park characteristics that can be readily applied across different urban contexts globally. The methodology provides a transferable framework for evaluating green space quality beyond simple area measurements, offering planners and policymakers evidence-based criteria for optimising mental health outcomes through strategic green infrastructure investment. We utilised robust, national spatial data quantifying the quantity of parks and their features, as well as surrounding UGS within three commonly used home catchments. We were able to conduct a sensitivity analysis that included several confounders that have been shown to be potentially associated with both health behaviours and outcomes, as well as parks.

The primary limitation of the study is that we did not know whether the sample utilised parks or, if they did so, what activities they undertook during their visits. We also used an assessment of park qualities that were important for human health but were unable to assess some characteristics of the parks, such as litter, graffiti, vandalism, perceived safety, and perceived quality. There were temporal differences between the spatial park level data and UK Biobank depression prevalence outcome measured at baseline; this is due to the quality of the park data not being sufficient at the earlier time periods. Further, the “ever been diagnosed” basis for the depression measure means that participants may have experienced and/or recovered from depression in an entirely different residential location. Biobank is also known to have sample bias, and the extent to which it represents the least advantaged in society is questionable. This study was cross-sectional, and we are unable to infer causation. Despite UK Biobank being a longitudinal cohort, the UGS measures and depression outcome were only measured at one timepoint, meaning a longitudinal analysis would have been inappropriate, and a longitudinal design would have been more appropriate to establish the causal inferences between greenspace exposure and mental health benefits. We encourage future longitudinal studies that examine proximity to parks and depression outcomes over the life course. Previous research has shown that multiple ascertainments are necessary to accurately estimate frequency of mental health conditions in UK Biobank, e.g. from health records and self-report [60]. In the absence of systematic, nationwide primary care linkage, analysis of green space metrics at baseline versus incident depression may be relatively underpowered and based solely on hospitalisations for depression, which are likely to be only the upper end in terms of severity. This may have its own limitations with regard to interpretation. Future research should investigate the role of greenspace on subsequent depression and other mental health condition incidence ideally with more systematic, thorough data linkage in UK Biobank (e.g. primary care). Our study highlights sex-based differences in our outcome of interest; in our sample, sex was recorded from the NHS record, asked at baseline assessment, and is based on biological sex at birth. There may be differences in the effects of both exposure and outcome for sex, as a biological construct, and gender, as a social construct. However, previous assessment of discordance between chromosomal and self-recorded sex in UK Biobank found only 200 discrepancies (< 0.04% of sample) [61], not allowing for meaningful analysis between groups. Our study population is constrained by UK Biobank’s recruitment design, which established a minimum age of 37 years [62]. This age threshold was deliberately chosen to optimise detection of incident common diseases and health outcomes while reducing confounding from competing mortality risks that increase substantially in older age groups.

## Conclusions

This large-scale analysis of 329,363 UK Biobank participants provides robust empirical evidence for sex-based differences in association between park characteristics and depression prevalence, with significant implications for urban planning practice internationally. Our findings demonstrate that park environments exhibit fundamentally different relationships with mental health outcomes for men and women, challenging current gender-neutral approaches to green space provision.

The findings provide cautious support for proximity-based planning concepts such as the 20-min neighbourhood framework, noting the sex-based differences we observed and extending beyond simple accessibility to identify specific features that optimise parks’ therapeutic potential for women. Our results suggest that current national design standards may be inadequate for maximising population mental health benefits, supporting extended catchment areas of 10–20-min walking distances, and prioritising amenities that facilitate social interaction and diverse recreational opportunities.

The methodology provides a transferable framework for evaluating green space quality in similar global contexts beyond simple area measurements, offering planners and policymakers internationally evidence-based criteria for optimising mental health outcomes. Future urban planning should adopt gender-informed approaches that recognise differential usage patterns, prioritising accessibility and integration of health-promoting amenities whilst investigating alternative pathways through which green spaces might support men’s mental health.

## Supplementary Information

Additional file 1: Table S1: Park characteristics, definition and source. Table S2a: Association between park, UGS variables and depression adjusted for health and behaviour outcomes (Females). Table S2b: Association between UGS variables and depression adjusted for health and behaviour outcomes (Males). Table S3: Sex-based interactions between mental health outcome and UGS variables. Table S4a: Sensitivity analysis with primary care depression diagnosis removed (Females). Table S4b: Sensitivity analysis with primary care depression diagnosis removed (Males). Table S5: Base models of covariates for stratified models.

## Data Availability

This research was conducted using the UK Biobank resource under access application 73613. All data created for this study have been provided to UK Biobank. UK Biobank will make the data available to bona fide researchers for all types of health-related research that is in the public interest. For more details on the access procedure, see the UK Biobank website: http://www.ukbiobank.ac.uk/register-apply

## Abbreviations

BAME: Black, Asian, and Minority Ethnic
BMI: Body mass index
CI: Confidence interval
GVIF: Generalised variance inflation factor
IMD: Index of Multiple Deprivation
M: Metres
OR: Odds ratio
SDI: Shannon diversity index
UGS: Urban green space
