# Supplementary material for: Characteristics of parks associated with depression in women only: a cross-sectional study of 329,363 adults
Source: BMC Med. 2026 Jan 21;24:98. doi: 10.1186/s12916-026-04641-1 (PMC12905948; doi:10.1186/s12916-026-04641-1)
Supplement: Supplementary file 1 — Additional file 1: Table S1: Park characteristics, definition and source. Table S2a: Association between park, UGS variables and depression adjusted for health and behaviour outcomes (Females). Table S2b: Association between UGS variables and depression adjusted for health and behaviour outcomes (Males). Table S3: Sex-based interactions between mental health outcome and UGS variables. Table S4a: Sensitivity analysis with primary care depression diagnosis removed (Females). Table S4b: Sensitivity analysis with primary care depression diagnosis removed (Males). Table S5: Base models of covariates for stratified models. [file 12916_2026_4641_MOESM1_ESM.docx]

**Characteristics of parks associated with depression in women only: a cross-sectional study of 329363 adults.**

**List of Supplementary Tables:**

Table S1: Park characteristics, definition and source.

Table S2a: Association between park, UGS variables and depression adjusted for health and behaviour outcomes (Females)

Table S2b: Association between UGS variables and depression adjusted for health and behaviour outcomes (Males)

Table S3: Sex-based interactions between mental health outcome and UGS variables

Table S4a: Sensitivity analysis with primary care depression diagnosis removed (Females).

Table S4b: Sensitivity analysis with primary care depression diagnosis removed (Males).

Table S5: Base models of covariates for stratified models

***Table S1: Park characteristics, definition and source.***

| **Variable** | | | **Definition** | **Source** |
| --- | --- | --- | --- | --- |
| **Home catchment area** | | | | |
| Catchment area | | 400m (10-min return walking distance) | Road and path network buffer isochrones were generated around each UK Biobank home location at 1600 metres (m), 800m, and 400m intervals to represent 20-minute, 10-minute, and 5-minute walking distances, respectively. The distances and associated walking times are commonly applied as suggested adult walking speeds^[[1]](#footnote-1)^ and are associated with local living and walkable neighbourhood policies, such as the 20-minute neighbourhood^[[2]](#footnote-2)^. OS OpenRoads data were used to generate the road and path network for Great Britain using the XY (Easting, Northing) coordinates of UK Biobank residents as origins. All origins were snapped to the closest network feature prior to analysis. | OS Open Roads (2020)^[[3]](#footnote-3)^ |
|  |  | 800m (20-min return walking distance) |  |  |
|  |  | 1600m (40-min return walking distance) |  |  |
| **Characteristics within parks** | | | | |
| Amenities | Café | | The number of parks with 1+ café (Class = 0461) or 1+ toilet (Class = 0013) within park and 100m for each catchment area. | Ordnance Survey Points of Interest (2020)^[[4]](#footnote-4)^ |
|  | Toilet | |  |  |
| Attractions | 1+ Attraction | | The number of parks with 1+ or 2+ public attractions within park and 100m for each catchment area. Public Attractions comprised features with the following classifications: ‘Botanical and zoological’ (Category=16), ‘Sightseeing, tours, viewing and visitor centres’ (Class=0267), ‘Laseria, observatories and planetaria’ (Class=0263), ‘Theme and adventure parks’ (Class=0266), ‘Model villages’ (Class=0264), ‘Unspecified and other attractions’ (Class=0269) and ‘Railways (heritage, steam and miniature)’ (Class=0265). | Ordnance Survey Points of Interest (2020) ^[[5]](#footnote-5)^ |
|  | 2+ Attraction | |  |  |
| Tree Cover | 20% of park area | | Forest and non-forest tree and woody vegetation data were derived from the CLMS datasets: 'Forest Type' (FTY): This dataset classifies forest cover in rural contexts as broadleaved (FTY = 1), coniferous (FTY = 2), or mixed (FTY = 3) at 10m resolution. The 'Forest Additional Support Layer' (FADSL) classifies forest cover in agricultural (broadleaved, FADSL = 3) and urban (broadleaved, FADSL = 4; coniferous, FADSL = 5) contexts at 10m resolution. The 'Small Woody Features' (SWF) dataset classifies non-forest tree cover and shorter woody vegetation such as hedgerows and bushes in all contexts at 5m resolution. Land Cover Map classifies broadleaf woodland (LCM = 1) and coniferous woodland (LCM = 2). | European Union's Copernicus Land Monitoring Service (CLMS)^[[6]](#footnote-6)^  Centre for Ecology & Hydrology Land Cover Map 2020^[[7]](#footnote-7)^ |
|  | 30% of park area | |  |  |
|  | 50% of park area | |  |  |
| Water feature | | | A ‘blue space’ layer from OS MasterMap was created for the UK. Marine water (sea and ocean) features were masked from the data and all other water body features. 'Water' features including both standing water (lakes, reservoirs, ponds) and moving water (rivers, streams, canals) features were included if situated within a park. | OS MasterMap (2020) Topography data^[[8]](#footnote-8)^ |
| Sports facilities | 1+ Sports facility | | The number of parks with 1+ or 2+ sports facilities within park perimeter. Defined as: Sports Complex: Category = 24; Outdoor Pursuits: Category = 23. | Ordnance Survey Points of Interest (2020) ^[[9]](#footnote-9)^ |
|  | 2+ Sports facility | |  |  |
| **Catchment park metrics** | | | | |
| Number of parks | | | Greenspace function was classed as a ‘Public Park or Garden’, defined as: ‘Areas of land designed, constructed, managed and maintained as a public park or garden. These normally have a defined perimeter and free public access and generally sit within or close to urban areas’. | OS Open Greenspace (2020)^[[10]](#footnote-10)^ |
| Number of large parks (> median 1.4ha) | | | Greenspace function was classed as a ‘Public Park or Garden’ over median 1.4ha size. |  |
| Area (m) of catchment covered by parks | | | Area of catchment (meters) consisting of park land use. |  |
| Proportion of catchment covered by parks | | | Proportion of total catchment area consisting of park land use. |  |
| **Urban Green Spaces (UGS) catchment metrics** | | | | |
| Mean size of UGS within catchment | | | UGS defined as accessible to the public, such as public parks, playing fields, sports facilities, play areas and allotments. Mean total area size of all UGS that are within catchment area. | OS Open Greenspace (2020) ^[[11]](#footnote-11)^ |
| Proportion of catchment UGS | | | Proportion of total catchment area consisting of UGS land use. |  |
| Proportion of UGS area classified as parks | | | Proportion of UGS land use within catchment defined as a public park. |  |
| Number of types of UGS | | | Number of types of UGS within catchment area. Types available: public parks, playing fields, sports facilities, play areas and allotments. |  |
| Shannon Diversity (SDI) of UGS types | | | Diversity of UGS types within catchment calculated using the Shannon Diversity Index (SDI). |  |
| **Non-catchment metrics** | | | | |
| Distance to nearest park | | | Distance to all parks was created as a single estimate of the network distance to the nearest park from each residential locations using QGIS Network Analysis Toolbox (Shortest Path Point to Layer tool) to calculate the most direct route along the road network infrastructure. Shortest distance extracted. | OS Open Greenspace (2020) ^[[12]](#footnote-12)^ |
| Mean distance to 10 nearest parks | | | Mean distance to 10 nearest parks using method stated above. |  |
| Mean distance to 10 nearest UGS | | | Distance to all UGS was created as a single estimate of the network distance to the nearest UGS from each residential locations using QGIS Network Analysis Toolbox (Shortest Path Point to Layer tool) to calculate the most direct route along the road network infrastructure.  Mean distance to 10 nearest UGS was generated using this calculation. |  |
| Distance to nearest park with a water feature | | | Method as stated above for parks that contained a water feature. Shortest distance extracted. |  |
| Distance to nearest Protected Area | | | UK and Scottish government that outlines the boundaries of Sites of Special Scientific Interest (SSSI). SSSI’s refer to land notified as an SSSI under the Wildlife and Countryside Act (1981), as amended. | Sites of Special Scientific Interest (England)^[[13]](#footnote-13)^  Site of Special Scientific Interest (Scotland) ^[[14]](#footnote-14)^ |

***Table S2a: Association between park, UGS variables and depression adjusted for health and behaviour outcomes (Females)***

| **Females** | | **400m catchment** | | | | **800m catchment** | | | | **1600m catchment** | | | | |
| --- | --- | --- | --- | --- | --- | --- | --- | --- | --- | --- | --- | --- | --- | --- |
|  |  | **OR** | **LCL** | **UCL** | **p** | **OR** | **LCL** | **UCL** | **p** | **OR** | **LCL** | **UCL** | **p** |  |
| Amenities | Café | 0.99 | 0.92 | 1.08 | 0.86 | 0.98 | 0.92 | 1.04 | 0.52 | 0.90 | 0.86 | 0.94 | <0.001 |  |
|  | Toilet | 1.02 | 0.94 | 1.10 | 0.67 | 0.96 | 0.90 | 1.02 | 0.17 | 0.88 | 0.81 | 0.94 | <0.001 |  |
| Attractions | 1+ attractions | 0.93 | 0.86 | 1.01 | 0.07 | 0.90 | 0.85 | 0.95 | 0.00 | 0.83 | 0.80 | 0.87 | <0.001 |  |
|  | 2+ attractions | 0.92 | 0.85 | 1.00 | 0.04 | 0.94 | 0.89 | 1.00 | 0.04 | 0.85 | 0.79 | 0.92 | <0.001 |  |
| Tree cover | 20% of park area | 1.00 | 0.92 | 1.09 | 0.92 | 0.94 | 0.89 | 1.00 | 0.03 | 0.89 | 0.86 | 0.92 | <0.001 |  |
|  | 30% of park area | 0.98 | 0.91 | 1.06 | 0.67 | 0.97 | 0.92 | 1.02 | 0.22 | 0.89 | 0.86 | 0.92 | <0.001 |  |
|  | 50% of park area | 0.96 | 0.90 | 1.04 | 0.33 | 0.96 | 0.91 | 1.01 | 0.14 | 0.88 | 0.84 | 0.91 | <0.001 |  |
| Water feature | | 1.02 | 0.94 | 1.09 | 0.69 | 1.01 | 0.96 | 1.07 | 0.70 | 0.87 | 0.82 | 0.93 | <0.001 |  |
| Sports facilities | 1+ sports facility | 0.99 | 0.91 | 1.06 | 0.69 | 0.96 | 0.91 | 1.02 | 0.20 | 0.84 | 0.79 | 0.90 | <0.001 |  |
|  | 2+ sports facility | 0.97 | 0.89 | 1.05 | 0.47 | 0.96 | 0.90 | 1.02 | 0.21 | 0.84 | 0.76 | 0.92 | <0.001 |  |
| Catchment park metrics | Number of parks | 0.99 | 0.91 | 1.07 | 0.73 | 0.98 | 0.96 | 1.00 | 0.06 | 0.98 | 0.97 | 0.98 | <0.001 |  |
|  | Number of large parks (> median 1.4ha) | 0.97 | 0.90 | 1.05 | 0.48 | 0.93 | 0.88 | 0.98 | 0.01 | 0.85 | 0.81 | 0.89 | <0.001 |  |
|  | Area (m) of catchment covered by parks | 1.00 | 1.00 | 1.00 | 0.83 | 1.00 | 1.00 | 1.00 | 0.15 | 1.00 | 1.00 | 1.00 | 0.005 |  |
|  | Proportion of catchment covered by parks | 1.21 | 0.77 | 1.88 | 0.41 | 0.77 | 0.52 | 1.13 | 0.19 | 0.37 | 0.25 | 0.53 | <0.001 |  |
| UGS catchment metrics | Mean size of UGS within catchment | 1.03 | 0.99 | 1.08 | 0.18 | 0.99 | 0.97 | 1.01 | 0.22 | 0.99 | 0.98 | 1.00 | 0.10 |  |
|  | Proportion of catchment UGS | 1.37 | 0.96 | 1.93 | 0.08 | 1.07 | 0.81 | 1.42 | 0.63 | 0.65 | 0.50 | 0.84 | 0.001 |  |
|  | Proportion of UGS area classified as parks | 0.91 | 0.81 | 1.02 | 0.09 | 0.89 | 0.82 | 0.95 | 0.00 | 0.76 | 0.71 | 0.82 | <0.001 |  |
|  | Number of types of UGS | 1.01 | 0.98 | 1.05 | 0.54 | 1.01 | 0.98 | 1.03 | 0.59 | 1.00 | 0.98 | 1.03 | 0.93 |  |
|  | Shannon Diversity of UGS types | 1.08 | 0.98 | 1.19 | 0.13 | 1.05 | 0.99 | 1.13 | 0.13 | 1.10 | 1.03 | 1.18 | 0.01 |  |
| **Non-catchment metrics** | | | | | | | | | | | | | | |
|  | | **OR** | | | **LL CI** | | | **UL CI** | | | **P** | | | |
| Distance to nearest park | | 1.02 | | | 1.01 | | | 1.03 | | | 0.01 | | | |
| Mean distance to 10 nearest parks | | 1.02 | | | 1.01 | | | 1.03 | | | <0.001 | | | |
| Mean distance to 10 nearest UGS | | 0.99 | | | 0.93 | | | 1.05 | | | 0.77 | | | |
| Distance to nearest park with a water feature | | 1.02 | | | 1.01 | | | 1.03 | | | <0.001 | | | |
| Distance to nearest Protected Area | | 1.01 | | | 1.00 | | | 1.02 | | | 0.01 | | | |

***Table S2b: Association between UGS variables and depression adjusted for health and behaviour outcomes (Males)***

| **Males** | | **400m catchment** | | | | **800m catchment** | | | | **1600m catchment** | | | | |
| --- | --- | --- | --- | --- | --- | --- | --- | --- | --- | --- | --- | --- | --- | --- |
|  |  | **OR** | **LCL** | **UCL** | **p** | **OR** | **LCL** | **UCL** | **p** | **OR** | **LCL** | **UCL** | **p** |  |
| Amenities | Café | 1.02 | 0.92 | 1.14 | 0.64 | 1.03 | 0.96 | 1.12 | 0.40 | 1.01 | 0.96 | 1.07 | 0.66 |  |
|  | Toilet | 1.06 | 0.95 | 1.18 | 0.30 | 1.10 | 1.01 | 1.19 | 0.02 | 1.08 | 0.98 | 1.18 | 0.12 |  |
| Attractions | 1+ attractions | 0.98 | 0.89 | 1.09 | 0.74 | 1.01 | 0.94 | 1.09 | 0.76 | 0.98 | 0.93 | 1.03 | 0.42 |  |
|  | 2+ attractions | 1.02 | 0.92 | 1.12 | 0.74 | 1.06 | 0.98 | 1.14 | 0.12 | 1.08 | 0.98 | 1.19 | 0.10 |  |
| Tree cover | 20% of park area | 1.02 | 0.91 | 1.14 | 0.78 | 1.01 | 0.93 | 1.09 | 0.84 | 0.97 | 0.93 | 1.00 | 0.08 |  |
|  | 30% of park area | 1.03 | 0.93 | 1.14 | 0.56 | 1.00 | 0.94 | 1.08 | 0.90 | 0.98 | 0.94 | 1.02 | 0.28 |  |
|  | 50% of park area | 1.01 | 0.92 | 1.11 | 0.83 | 1.00 | 0.93 | 1.07 | 0.98 | 0.96 | 0.91 | 1.01 | 0.13 |  |
| Water feature | | 1.11 | 1.00 | 1.22 | 0.04 | 1.04 | 0.97 | 1.12 | 0.24 | 1.04 | 0.96 | 1.13 | 0.31 |  |
| Sports facilities | 1+ sports facility | 0.94 | 0.85 | 1.03 | 0.20 | 1.08 | 1.00 | 1.16 | 0.05 | 1.05 | 0.97 | 1.14 | 0.25 |  |
|  | 2+ sports facility | 0.99 | 0.89 | 1.11 | 0.92 | 1.06 | 0.98 | 1.15 | 0.17 | 1.08 | 0.96 | 1.22 | 0.19 |  |
| Park metrics | Number of parks | 0.95 | 0.86 | 1.06 | 0.37 | 0.99 | 0.96 | 1.02 | 0.49 | 0.99 | 0.99 | 1.00 | 0.12 |  |
|  | Number of large parks (> median 1.4ha) | 1.13 | 1.02 | 1.25 | 0.02 | 1.01 | 0.94 | 1.09 | 0.75 | 0.98 | 0.92 | 1.04 | 0.44 |  |
|  | Area (m) of catchment covered by parks | 1.00 | 1.00 | 1.00 | 0.05 | 1.00 | 1.00 | 1.00 | 0.11 | 1.00 | 1.00 | 1.00 | 0.84 |  |
|  | Proportion of catchment covered by parks | 1.61 | 0.91 | 2.77 | 0.10 | 1.25 | 0.76 | 2.03 | 0.38 | 0.92 | 0.57 | 1.48 | 0.74 |  |
| UGS metrics | Mean size of UGS within catchment | 1.04 | 0.98 | 1.10 | 0.18 | 1.01 | 0.99 | 1.03 | 0.26 | 0.99 | 0.98 | 1.01 | 0.26 |  |
|  | Proportion of catchment UGS | 1.35 | 0.85 | 2.12 | 0.20 | 1.24 | 0.86 | 1.78 | 0.26 | 0.96 | 0.68 | 1.35 | 0.81 |  |
|  | Proportion of UGS area classified as parks | 1.16 | 1.00 | 1.34 | 0.06 | 0.99 | 0.89 | 1.09 | 0.80 | 0.98 | 0.89 | 1.08 | 0.64 |  |
|  | Number of types of UGS | 0.99 | 0.95 | 1.04 | 0.68 | 1.01 | 0.98 | 1.04 | 0.58 | 1.01 | 0.97 | 1.04 | 0.71 |  |
|  | Shannon Diversity of UGS types | 0.98 | 0.86 | 1.11 | 0.70 | 0.98 | 0.90 | 1.08 | 0.73 | 1.04 | 0.95 | 1.14 | 0.39 |  |
| **Non-catchment metrics** | | | | | | | | | | | | | | |
|  | | **OR** | | | **LL CI** | | | **UL CI** | | | **P** | | | |
| Distance to nearest park | | 0.993 | | | 0.97 | | | 1.01 | | | 0.43 | | | |
| Mean distance to 10 nearest parks | | 0.996 | | | 0.98 | | | 1.01 | | | 0.58 | | | |
| Mean distance to 10 nearest UGS | | 0.953 | | | 0.88 | | | 1.03 | | | 0.23 | | | |
| Distance to nearest park with a water feature | | 0.997 | | | 0.98 | | | 1.01 | | | 0.70 | | | |
| Distance to nearest Protected Area | | 1.004 | | | 0.99 | | | 1.02 | | | 0.49 | | | |

***Table S3: Sex-based interactions between mental health outcome and UGS variables***

| **Sex-based Interaction effects** | | **400m catchment** | | | | **800m catchment** | | | | **1600m catchment** | | | | |
| --- | --- | --- | --- | --- | --- | --- | --- | --- | --- | --- | --- | --- | --- | --- |
|  |  | **beta** | **se** | **z value** | **Pr (>\|z\|)** | **beta** | **se** | **z value** | **Pr (>\|z\|)** | **beta** | **se** | **z value** | **Pr (>\|z\|)** |  |
| Parks within catchment | | 0.09 | 0.03 | 2.58 | 0.01 | 0.10 | 0.03 | 3.72 | <0.001 | 0.06 | 0.03 | 1.84 | 0.07 |  |
| Amenities | Café | 0.06 | 0.07 | 0.85 | 0.40 | 0.07 | 0.05 | 1.49 | 0.14 | 0.13 | 0.03 | 3.66 | <0.001 |  |
|  | Toilet | 0.05 | 0.07 | 0.71 | 0.48 | 0.14 | 0.05 | 2.77 | 0.01 | 0.23 | 0.06 | 3.96 | <0.001 |  |
| Attractions | 1+ attractions | 0.06 | 0.06 | 0.94 | 0.35 | 0.12 | 0.05 | 2.62 | 0.01 | 0.16 | 0.03 | 4.80 | <0.001 |  |
|  | 2+ attractions | 0.11 | 0.06 | 1.67 | 0.10 | 0.13 | 0.05 | 2.65 | 0.01 | 0.25 | 0.06 | 4.29 | <0.001 |  |
| Tree cover | 20% of park area | 0.02 | 0.07 | 0.26 | 0.79 | 0.08 | 0.05 | 1.54 | 0.12 | 0.09 | 0.03 | 3.64 | <0.001 |  |
|  | 30% of park area | 0.05 | 0.06 | 0.81 | 0.42 | 0.04 | 0.04 | 0.91 | 0.36 | 0.11 | 0.03 | 3.88 | <0.001 |  |
|  | 50% of park area | 0.05 | 0.06 | 0.79 | 0.43 | 0.04 | 0.05 | 0.92 | 0.36 | 0.10 | 0.03 | 2.99 | 0.003 |  |
| Water feature | | 0.08 | 0.06 | 1.24 | 0.21 | 0.03 | 0.04 | 0.58 | 0.56 | 0.18 | 0.05 | 3.57 | <0.001 |  |
| Sports facilities | 1+ sports facility | -0.03 | 0.06 | -0.47 | 0.64 | 0.12 | 0.05 | 2.64 | 0.01 | 0.23 | 0.05 | 4.36 | <0.001 |  |
|  | 2+ sports facility | 0.04 | 0.07 | 0.57 | 0.57 | 0.11 | 0.05 | 2.09 | 0.04 | 0.27 | 0.08 | 3.60 | <0.001 |  |
| Park metrics | Number of parks | -0.02 | 0.07 | -0.37 | 0.71 | 0.02 | 0.02 | 1.01 | 0.31 | 0.02 | 0.00 | 4.04 | <0.001 |  |
|  | Number of large parks (> median 1.4ha) | 0.15 | 0.07 | 2.19 | 0.03 | 0.08 | 0.05 | 1.73 | 0.08 | 0.15 | 0.04 | 3.82 | <0.001 |  |
|  | Area (m) of catchment covered by parks | 0.00 | 0.00 | 1.41 | 0.16 | 0.00 | 0.00 | 2.24 | 0.03 | 0.98 | 0.31 | 3.18 | 0.001 |  |
|  | Proportion of catchment covered by parks | 0.27 | 0.36 | 0.73 | 0.46 | 0.52 | 0.32 | 1.63 | 0.10 | 0.00 | 0.00 | 1.94 | 0.05 |  |
| UGS metrics | Mean size of UGS within catchment | 0.01 | 0.04 | 0.14 | 0.89 | 0.02 | 0.01 | 1.48 | 0.14 | 0.00 | 0.01 | -0.01 | 0.99 |  |
|  | Proportion of catchment UGS | -0.01 | 0.29 | -0.05 | 0.96 | 0.17 | 0.24 | 0.74 | 0.46 | 0.44 | 0.22 | 2.01 | 0.04 |  |
|  | Proportion of UGS area classified as parks | 0.22 | 0.09 | 2.39 | 0.02 | 0.10 | 0.06 | 1.64 | 0.10 | 0.26 | 0.06 | 4.16 | <0.001 |  |
|  | Number of types of UGS | -0.01 | 0.03 | -0.41 | 0.68 | 0.01 | 0.02 | 0.58 | 0.56 | 0.01 | 0.02 | 0.53 | 0.60 |  |
|  | Shannon Diversity of UGS types | -0.08 | 0.08 | -0.96 | 0.34 | -0.05 | 0.06 | -0.82 | 0.41 | -0.05 | 0.06 | -0.86 | 0.39 |  |
| **Non-catchment metrics** | | | | | | | | | | | | | | |
|  | | **beta** | | | **se** | | | **z value** | | | **Pr (>\|z\|)** | | | |
| Distance to nearest park | | -0.03 | | | 0.01 | | | -2.42 | | | 0.02 | | | |
| Mean distance to 10 nearest parks | | -0.03 | | | 0.01 | | | -3.02 | | | 0.003 | | | |
| Mean distance to 10 nearest UGS | | -0.05 | | | 0.05 | | | -1.12 | | | 0.26 | | | |
| Distance to nearest park with a water feature | | -0.02 | | | 0.01 | | | -2.89 | | | 0.004 | | | |
| Distance to nearest Protected Area | | -0.01 | | | 0.01 | | | -0.83 | | | 0.40 | | | |

***Table S4a: Sensitivity analysis with primary care depression diagnosis removed (Females).***

| **Females** | | **400m catchment** | | | | **800m catchment** | | | | **1600m catchment** | | | | |
| --- | --- | --- | --- | --- | --- | --- | --- | --- | --- | --- | --- | --- | --- | --- |
|  |  | **OR** | **LCL** | **UCL** | **p** | **OR** | **LCL** | **UCL** | **p** | **OR** | **LCL** | **UCL** | **p** |  |
| Amenities | Café | 0.98 | 0.90 | 1.07 | 0.72 | 0.94 | 0.88 | 1.00 | 0.07 | 0.91 | 0.86 | 0.95 | <0.001 |  |
|  | Toilet | 1.06 | 0.97 | 1.15 | 0.23 | 0.98 | 0.92 | 1.05 | 0.60 | 0.92 | 0.86 | 1.00 | 0.05 |  |
| Attractions | 1+ attractions | 0.94 | 0.86 | 1.02 | 0.12 | 0.92 | 0.86 | 0.97 | 0.004 | 0.89 | 0.85 | 0.93 | <0.001 |  |
|  | 2+ attractions | 0.95 | 0.87 | 1.03 | 0.21 | 0.96 | 0.90 | 1.02 | 0.18 | 0.90 | 0.83 | 0.97 | 0.01 |  |
| Tree cover | 20% of park area | 0.97 | 0.89 | 1.07 | 0.56 | 0.91 | 0.86 | 0.97 | 0.00 | 0.92 | 0.89 | 0.95 | <0.001 |  |
|  | 30% of park area | 0.95 | 0.88 | 1.03 | 0.25 | 0.94 | 0.88 | 0.99 | 0.03 | 0.92 | 0.88 | 0.95 | <0.001 |  |
|  | 50% of park area | 1.00 | 0.92 | 1.08 | 0.92 | 0.98 | 0.92 | 1.03 | 0.41 | 0.91 | 0.87 | 0.96 | <0.001 |  |
| Water feature | | 1.01 | 0.93 | 1.09 | 0.82 | 1.01 | 0.95 | 1.07 | 0.72 | 0.90 | 0.84 | 0.97 | 0.003 |  |
| Sports facilities | 1+ sports facility | 0.98 | 0.90 | 1.06 | 0.55 | 0.95 | 0.90 | 1.01 | 0.11 | 0.88 | 0.82 | 0.94 | <0.001 |  |
|  | 2+ sports facility | 0.99 | 0.90 | 1.08 | 0.77 | 0.98 | 0.91 | 1.05 | 0.48 | 0.91 | 0.83 | 1.01 | 0.08 |  |
| Catchment park metrics | Number of parks | 1.00 | 0.92 | 1.09 | 0.95 | 0.99 | 0.97 | 1.02 | 0.60 | 0.99 | 0.98 | 0.99 | <0.001 |  |
|  | Number of large parks (> median 1.4ha) | 0.99 | 0.91 | 1.08 | 0.77 | 0.93 | 0.87 | 0.98 | 0.01 | 0.90 | 0.86 | 0.95 | <0.001 |  |
|  | Area (m) of catchment covered by parks | 1.00 | 1.00 | 1.00 | 0.67 | 1.00 | 1.00 | 1.00 | 0.17 | 1.00 | 1.00 | 1.00 | 0.055 |  |
|  | Proportion of catchment covered by parks | 1.29 | 0.79 | 2.08 | 0.30 | 0.83 | 0.54 | 1.26 | 0.39 | 0.50 | 0.34 | 0.75 | 0.001 |  |
| UGS catchment metrics | Mean size of UGS within catchment | 1.03 | 0.97 | 1.08 | 0.32 | 0.99 | 0.97 | 1.01 | 0.28 | 1.00 | 0.98 | 1.01 | 0.53 |  |
|  | Proportion of catchment UGS | 1.34 | 0.91 | 1.95 | 0.14 | 1.10 | 0.81 | 1.49 | 0.54 | 0.80 | 0.60 | 1.06 | 0.118 |  |
|  | Proportion of UGS area classified as parks | 0.95 | 0.85 | 1.08 | 0.45 | 0.90 | 0.83 | 0.98 | 0.01 | 0.83 | 0.77 | 0.90 | <0.001 |  |
|  | Number of types of UGS | 1.01 | 0.97 | 1.05 | 0.61 | 1.01 | 0.98 | 1.03 | 0.58 | 1.01 | 0.98 | 1.04 | 0.48 |  |
|  | Shannon Diversity of UGS types | 1.05 | 0.94 | 1.17 | 0.40 | 1.03 | 0.96 | 1.11 | 0.42 | 1.06 | 0.99 | 1.14 | 0.11 |  |
| **Non-catchment metrics** | | | | | | | | | | | | | | |
|  | | **OR** | | | **LL CI** | | | **UL CI** | | | **P** | | | |
| Distance to nearest park | | 1.01 | | | 1.00 | | | 1.03 | | | 0.13 | | | |
| Mean distance to 10 nearest parks | | 1.01 | | | 1.00 | | | 1.02 | | | 0.04 | | | |
| Mean distance to 10 nearest UGS | | 1.00 | | | 0.94 | | | 1.07 | | | 0.96 | | | |
| Distance to nearest park with a water feature | | 1.01 | | | 1.00 | | | 1.02 | | | 0.05 | | | |
| Distance to nearest Protected Area | | 1.01 | | | 1.01 | | | 1.02 | | | 0.002 | | | |

***Table S4b: Sensitivity analysis with primary care depression diagnosis removed (Males).***

| **Males** | | **400m catchment** | | | | **800m catchment** | | | | **1600m catchment** | | | | |
| --- | --- | --- | --- | --- | --- | --- | --- | --- | --- | --- | --- | --- | --- | --- |
|  |  | **OR** | **LCL** | **UCL** | **p** | **OR** | **LCL** | **UCL** | **p** | **OR** | **LCL** | **UCL** | **p** |  |
| Amenities | Café | 1.03 | 0.92 | 1.15 | 0.61 | 1.03 | 0.95 | 1.12 | 0.50 | 1.05 | 0.99 | 1.11 | 0.13 |  |
|  | Toilet | 1.04 | 0.93 | 1.17 | 0.46 | 1.09 | 1.00 | 1.19 | 0.05 | 1.12 | 1.01 | 1.23 | 0.03 |  |
| Attractions | 1+ attractions | 1.00 | 0.89 | 1.12 | 0.98 | 1.03 | 0.95 | 1.12 | 0.43 | 1.04 | 0.98 | 1.10 | 0.23 |  |
|  | 2+ attractions | 1.05 | 0.94 | 1.17 | 0.40 | 1.08 | 1.00 | 1.17 | 0.05 | 1.13 | 1.02 | 1.25 | 0.02 |  |
| Tree cover | 20% of park area | 1.02 | 0.90 | 1.15 | 0.76 | 1.00 | 0.92 | 1.08 | 0.93 | 1.00 | 0.96 | 1.04 | 1.00 |  |
|  | 30% of park area | 1.01 | 0.91 | 1.13 | 0.81 | 0.98 | 0.90 | 1.05 | 0.52 | 1.00 | 0.96 | 1.05 | 0.87 |  |
|  | 50% of park area | 1.04 | 0.94 | 1.16 | 0.46 | 0.99 | 0.92 | 1.07 | 0.87 | 1.00 | 0.95 | 1.06 | 0.97 |  |
| Water feature | | 1.15 | 1.04 | 1.28 | 0.01 | 1.07 | 1.00 | 1.16 | 0.06 | 1.09 | 1.00 | 1.19 | 0.04 |  |
| Sports facilities | 1+ sports facility | 0.94 | 0.85 | 1.05 | 0.26 | 1.10 | 1.02 | 1.19 | 0.02 | 1.09 | 1.00 | 1.19 | 0.06 |  |
|  | 2+ sports facility | 1.05 | 0.93 | 1.17 | 0.46 | 1.11 | 1.01 | 1.21 | 0.03 | 1.16 | 1.02 | 1.32 | 0.03 |  |
| Park metrics | Number of parks | 0.97 | 0.87 | 1.08 | 0.61 | 1.01 | 0.98 | 1.03 | 0.72 | 1.00 | 1.00 | 1.01 | 0.44 |  |
|  | Number of large parks (> median 1.4ha) | 1.12 | 1.00 | 1.26 | 0.05 | 1.04 | 0.96 | 1.12 | 0.39 | 1.03 | 0.97 | 1.10 | 0.35 |  |
|  | Area (m) of catchment covered by parks | 1.00 | 1.00 | 1.00 | 0.20 | 1.00 | 1.00 | 1.00 | 0.15 | 1.00 | 1.00 | 1.00 | 0.43 |  |
|  | Proportion of catchment covered by parks | 1.39 | 0.74 | 2.54 | 0.31 | 1.36 | 0.80 | 2.29 | 0.26 | 1.35 | 0.80 | 2.24 | 0.25 |  |
| UGS metrics | Mean size of UGS within catchment | 1.02 | 0.96 | 1.09 | 0.49 | 1.01 | 0.99 | 1.03 | 0.44 | 0.99 | 0.98 | 1.01 | 0.39 |  |
|  | Proportion of catchment UGS | 1.01 | 0.60 | 1.67 | 0.97 | 1.26 | 0.84 | 1.87 | 0.26 | 1.15 | 0.79 | 1.66 | 0.47 |  |
|  | Proportion of UGS area classified as parks | 1.22 | 1.03 | 1.43 | 0.02 | 0.99 | 0.88 | 1.10 | 0.80 | 1.08 | 0.97 | 1.20 | 0.16 |  |
|  | Number of types of UGS | 0.99 | 0.94 | 1.04 | 0.67 | 1.02 | 0.98 | 1.05 | 0.35 | 1.01 | 0.97 | 1.05 | 0.62 |  |
|  | Shannon Diversity of UGS types | 1.00 | 0.87 | 1.15 | 0.99 | 0.99 | 0.90 | 1.09 | 0.85 | 1.04 | 0.95 | 1.15 | 0.38 |  |
| **Non-catchment metrics** | | | | | | | | | | | | | | |
|  | | **OR** | | | **LL CI** | | | **UL CI** | | | **P** | | | |
| Distance to nearest park | | 0.987 | | | 0.97 | | | 1.01 | | | 0.19 | | | |
| Mean distance to 10 nearest parks | | 0.987 | | | 0.97 | | | 1.00 | | | 0.14 | | | |
| Mean distance to 10 nearest UGS | | 0.967 | | | 0.89 | | | 1.05 | | | 0.45 | | | |
| Distance to nearest park with a water feature | | 0.989 | | | 0.97 | | | 1.00 | | | 0.13 | | | |
| Distance to nearest Protected Area | | 1.006 | | | 0.99 | | | 1.02 | | | 0.36 | | | |

***Table S5: Base models of covariates for stratified models***

| Base Sample | | **400m** | | | | **800m** | | | | **1600m** | | | |
| --- | --- | --- | --- | --- | --- | --- | --- | --- | --- | --- | --- | --- | --- |
|  |  | **beta** | **se** | **z value** | **Pr(>\|z\|)** | **beta** | **se** | **z value** | **Pr(>\|z\|)** | **beta** | **se** | **z value** | **Pr(>\|z\|)** |
| **(a) Female** | | | | | | | | | | | | | |
| Intercept | | -0.32 | 0.18 | -1.76 | 0.08 | -0.36 | 0.12 | -3.08 | 0.00 | -0.34 | 0.09 | -3.74 | <0.001 |
| Age (Median (IQR)) | | -0.03 | 0.00 | -8.62 | <0.001 | -0.03 | 0.00 | -13.05 | <0.001 | -0.03 | 0.00 | -16.99 | <0.001 |
| Area-level income deprivation (quintiles) | Q1 - least deprived | *Ref* | | | | *Ref* | | | | *Ref* | | | |
|  | Q2 | -0.09 | 0.07 | -1.24 | 0.21 | -0.01 | 0.04 | -0.29 | 0.77 | 0.06 | 0.03 | 1.87 | 0.06 |
|  | Q3 | 0.01 | 0.06 | 0.21 | 0.84 | 0.01 | 0.04 | 0.29 | 0.77 | 0.06 | 0.03 | 2.20 | 0.03 |
|  | Q4 | -0.02 | 0.06 | -0.31 | 0.76 | 0.00 | 0.04 | -0.08 | 0.93 | 0.08 | 0.03 | 2.62 | 0.01 |
|  | Q5 - most deprived | -0.03 | 0.06 | -0.47 | 0.64 | 0.01 | 0.04 | 0.35 | 0.725 | 0.11 | 0.03 | 3.58 | <0.001 |
| Ethnicity | White | *Ref* | | | | *Ref* | | | | *Ref* | | | |
|  | BAME | -0.80 | 0.10 | -8.31 | <0.001 | -0.71 | 0.06 | -11.83 | <0.001 | -0.74 | 0.05 | -15.04 | <0.001 |
| Household income (£) | Less than 18,000 | *Ref* | | | | *Ref* | | | | *Ref* | | | |
|  | 18,001 to 30,999 | -0.31 | 0.05 | -6.02 | <0.001 | -0.33 | 0.03 | -10.20 | <0.001 | -0.31 | 0.03 | -12.30 | <0.001 |
|  | 31,000 to 51,999 | -0.51 | 0.05 | -9.34 | <0.001 | -0.56 | 0.03 | -16.14 | <0.001 | -0.55 | 0.03 | -20.12 | <0.001 |
|  | 52,000 to 99,999 | -0.84 | 0.06 | -12.98 | <0.001 | -0.87 | 0.04 | -20.91 | <0.001 | -0.87 | 0.03 | -26.86 | <0.001 |
|  | More than 100,000 | -0.94 | 0.10 | -9.39 | <0.001 | -1.05 | 0.07 | -15.72 | <0.001 | -1.10 | 0.06 | -20.03 | <0.001 |
| Education | Higher Education | *Ref* | | | | *Ref* | | | | *Ref* | | | |
|  | Other | -0.05 | 0.04 | -1.35 | 0.176 | -0.05 | 0.02 | -2.07 | 0.04 | -0.07 | 0.02 | -3.75 | <0.001 |
| Employment status | Employed | *Ref* | | | | *Ref* | | | | *Ref* | | | |
|  | Retired | 0.05 | 0.06 | 0.85 | 0.39 | 0.02 | 0.04 | 0.61 | 0.54 | 0.02 | 0.03 | 0.55 | 0.58 |
|  | Other | 0.12 | 0.08 | 1.44 | 0.15 | 0.14 | 0.05 | 2.68 | 0.007 | 0.14 | 0.04 | 3.50 | <0.001 |
|  | Unable to work | 1.47 | 0.08 | 17.28 | <0.001 | 1.43 | 0.05 | 26.20 | <0.001 | 1.47 | 0.04 | 33.84 | <0.001 |
|  | Unemployed | 0.29 | 0.15 | 1.92 | 0.05 | 0.45 | 0.09 | 4.82 | <0.001 | 0.40 | 0.08 | 5.32 | <0.001 |
| **(b) Male** | | | | | | | | | | | | | |
| Intercept | | -0.55 | 0.23 | -2.38 | 0.02 | -0.95 | 0.15 | -6.38 | <0.001 | -1.06 | 0.12 | -9.02 | <0.001 |
| Age (Median (IQR)) | | -0.03 | 0.00 | -8.03 | <0.001 | -0.02 | 0.00 | -9.47 | <0.001 | -0.02 | 0.00 | -11.32 | <0.001 |
| Area-level income deprivation (quintiles) | Q1 - least deprived | *Ref* | | | | *Ref* | | | | *Ref* | | | |
|  | Q2 | 0.03 | 0.09 | 0.28 | 0.78 | 0.05 | 0.06 | 0.81 | 0.42 | 0.05 | 0.04 | 1.30 | 0.19 |
|  | Q3 | 0.14 | 0.08 | 1.75 | 0.08 | 0.11 | 0.05 | 2.15 | 0.03 | 0.13 | 0.04 | 3.28 | 0.00 |
|  | Q4 | 0.04 | 0.08 | 0.44 | 0.66 | 0.06 | 0.05 | 1.17 | 0.24 | 0.11 | 0.04 | 2.79 | 0.01 |
|  | Q5 - most deprived | 0.06 | 0.08 | 0.79 | 0.43 | 0.10 | 0.05 | 1.95 | 0.051 | 0.11 | 0.04 | 2.80 | 0.01 |
| Ethnicity | White | *Ref* | | | | *Ref* | | | | *Ref* | | | |
|  | BAME | -0.82 | 0.12 | -6.65 | <0.001 | -0.74 | 0.08 | -9.24 | <0.001 | -0.73 | 0.07 | -10.86 | <0.001 |
| Household income (£) | Less than 18,000 | *Ref* | | | | *Ref* | | | | *Ref* | | | |
|  | 18,001 to 30,999 | -0.33 | 0.07 | -4.54 | <0.001 | -0.35 | 0.05 | -7.43 | <0.001 | -0.35 | 0.04 | -9.44 | <0.001 |
|  | 31,000 to 51,999 | -0.55 | 0.08 | -7.16 | <0.001 | -0.55 | 0.05 | -11.08 | <0.001 | -0.53 | 0.04 | -13.51 | <0.001 |
|  | 52,000 to 99,999 | -0.87 | 0.09 | -9.82 | <0.001 | -0.89 | 0.06 | -15.41 | <0.001 | -0.87 | 0.05 | -19.15 | <0.001 |
|  | More than 100,000 | -1.23 | 0.13 | -9.13 | <0.001 | -1.29 | 0.09 | -14.06 | <0.001 | -1.18 | 0.07 | -16.32 | <0.001 |
| Education | Higher Education | *Ref* | | | | *Ref* | | | | *Ref* | | | |
|  | Other | -0.31 | 0.05 | -6.00 | <0.001 | -0.29 | 0.03 | -8.70 | <0.001 | -0.26 | 0.03 | -9.80 | <0.001 |
| Employment status | Employed | *Ref* | | | | *Ref* | | | | *Ref* | | | |
|  | Retired | 0.29 | 0.08 | 3.88 | <0.001 | 0.04 | 0.04 | 1.14 | 0.26 | 0.17 | 0.04 | 4.45 | <0.001 |
|  | Other | 0.79 | 0.16 | 4.91 | <0.001 | 0.16 | 0.05 | 3.12 | 0.002 | 0.62 | 0.09 | 6.58 | <0.001 |
|  | Unable to work | 1.74 | 0.09 | 18.71 | <0.001 | 1.29 | 0.06 | 23.19 | <0.001 | 1.71 | 0.05 | 34.26 | <0.001 |
|  | Unemployed | 0.32 | 0.14 | 2.32 | 0.02 | 0.40 | 0.09 | 4.31 | <0.001 | 0.32 | 0.07 | 4.32 | <0.001 |

1. Barton, Hugh, Marcus Grant, and Richard Guise. Shaping neighbourhoods: a guide for health, sustainability and vitality. Taylor & Francis, 2003. [↑](#footnote-ref-1)
2. Thornton, Lukar E., et al. "Operationalising the 20-minute neighbourhood." International Journal of Behavioral Nutrition and Physical Activity 19.1 (2022): 1-18. [↑](#footnote-ref-2)
3. <https://beta.ordnancesurvey.co.uk/products/os-open-roads> [↑](#footnote-ref-3)
4. <https://beta.ordnancesurvey.co.uk/products/points-of-interest> [↑](#footnote-ref-4)
5. <https://beta.ordnancesurvey.co.uk/products/points-of-interest> [↑](#footnote-ref-5)
6. <https://land.copernicus.eu/pan-european/high-resolution-layers> [↑](#footnote-ref-6)
7. <https://www.ceh.ac.uk/data/ukceh-land-cover-maps> [↑](#footnote-ref-7)
8. <https://beta.ordnancesurvey.co.uk/products/os-mastermap-topography-layer> [↑](#footnote-ref-8)
9. <https://beta.ordnancesurvey.co.uk/products/points-of-interest> [↑](#footnote-ref-9)
10. <https://beta.ordnancesurvey.co.uk/products/os-open-greenspace> [↑](#footnote-ref-10)
11. <https://beta.ordnancesurvey.co.uk/products/os-open-greenspace> [↑](#footnote-ref-11)
12. <https://beta.ordnancesurvey.co.uk/products/os-open-greenspace> [↑](#footnote-ref-12)
13. <https://www.data.gov.uk/dataset/5b632bd7-9838-4ef2-9101-ea9384421b0d/sites-of-special-scientific-interest-england> [↑](#footnote-ref-13)
14. <https://www.data.gov.uk/dataset/d64bf689-4ce8-465b-b00e-6a57dec94a22/site-of-special-scientific-interest-scotland> [↑](#footnote-ref-14)
